# Safety and prognostic value of left ventricular endomyocardial biopsy in dilated cardiomyopathy

**DOI:** 10.1002/ejhf.70019

**Published:** 2025-09-19

**Authors:** Elham Kayvanpour, Farbod Sedaghat‐Hamedani, Daniel Tian Li, Ebe Amr, Ali Amr, Bernd Lahrmann, Alan Lai, Christoph Reich, Chenyang Wang, Esther Herpel, Derliz Mereles, Lutz Frankenstein, Niels Grabe, Hugo A. Katus, Norbert Frey, Benjamin Meder

**Affiliations:** ^1^ Department of Medicine III University of Heidelberg Heidelberg Germany; ^2^ DZHK (German Centre for Cardiovascular Research) Heidelberg Germany; ^3^ Hamamatsu Tissue Imaging and Analysis Center (BIOQUANT) Heidelberg Germany; ^4^ Institute of Pathology University Medical Center Göttingen UMG Göttingen Germany; ^5^ Roche Diagnostics GmbH Mannheim Germany; ^6^ Tissue Bank of the National Center for Tumor Diseases (NCT) Heidelberg Germany; ^7^ Center of Clinical Pathology Klinikum Darmstadt Darmstadt Germany; ^8^ Department of Genetics, Stanford Genome Technology Center Stanford University School of Medicine Stanford CA USA

**Keywords:** Dilated cardiomyopathy, Left ventricular endomyocardial biopsy, Collagen volume fraction, Outcome

## Abstract

**Aims:**

The need to perform endomyocardial biopsy (EMB) in patients with non‐ischaemic dilated cardiomyopathies (DCM) is debated. Here we sought to determine the extent of left ventricular collagen volume fraction (LV‐CVF) in DCM patients and to evaluate it as a prognostic marker.

**Methods and results:**

In this retrospective longitudinal study, we included 524 patients with suspected DCM who underwent left ventricular EMB (LV‐EMB) as a part of their clinical work‐up. LV‐CVF was quantified using automated image processing of high‐resolution scans of LV‐EMB. Deep phenotyping was performed including assessment of late gadolinium enhancement on cardiac magnetic resonance imaging. Endpoints were (i) composite endpoint of heart failure‐related death, sudden cardiac death, aborted sudden cardiac death (appropriate implantable cardioverter‐defibrillator shock, reported sustained ventricular tachycardia, or cardiopulmonary resuscitation), or cardiac transplantation, and (ii) all‐cause mortality. LV‐EMB was associated with 0.76% major and 2.1% minor complications. No death occurred due to EMB. LV‐CVF could be reliably quantified using Bayesian classification. During a median follow‐up of 43.2 months (2084 patient‐years), 48 patients with LV‐CVF >32% and 14 patients with LV‐CVF ≤32% reached the composite endpoint (log‐rank *p* < 0.0001). A total of 62 patients reached the endpoint all‐cause mortality, from which 38 presented with LV‐CVF >32% and 17 with LV‐CVF ≤32% (log‐rank *p* = 0.009). In multivariable analyses, LV‐CVF and N‐terminal pro‐B‐type natriuretic peptide (NT‐proBNP) (hazard ratio 2.03, 95% confidence interval 1.32–3.11) were independent predictors of unfavourable outcome.

**Conclusions:**

Left ventricular EMB is a safe diagnostic procedure. The extent of CVF in LV‐EMB provides prognostic information in patients with DCM in addition to existing measures of left ventricular ejection fraction or NT‐proBNP.

## Introduction

Non‐ischaemic dilated cardiomyopathy (DCM), clinically defined as left ventricular or biventricular enlargement with decreased systolic function, not otherwise explained by abnormal loading conditions for example hypertension, valvular, or coronary artery disorders, is responsible for a significant proportion of newly diagnosed heart failure (HF) cases. DCM can lead to sudden cardiac death (SCD) or death as a result of progressive HF.^1^ In the workup of DCM, left heart catheterization is used to exclude coronary artery disease and optionally assess left ventricular endomyocardial biopsies (LV‐EMB) to aid differential diagnosis.

The most common indication for EMB is monitoring heart transplant rejection. Additionally, EMB can particularly complement diagnostics in specific clinical scenarios, such as cardiac sarcoidosis, amyloidosis, infectious/inflammatory cardiomyopathies, storage diseases like haemochromatosis, cardiac tumours, and drug‐related cardiotoxicity, allowing for tailored management strategies for these conditions.^2^ In cases of suspected giant‐cell myocarditis, necrotizing eosinophilic myocarditis, and immune checkpoint inhibitor myocarditis, EMB is often the definitive diagnostic tool, as clinical presentations and non‐invasive tests can be non‐specific.^3^ Accurate diagnosis in these cases is essential for initiating appropriate treatment, whether it involves immunosuppressive therapy or supportive care.

Due to the absence of randomized clinical studies, recommendations for EMB are currently based on retrospective analyses, case series, and expert opinions. The latest expert consensus from the Heart Failure Association, Heart Failure Society of America, and Japanese Heart Failure Society suggests, besides the aforementioned scenarios, performing EMB in patients with DCM in the context of decompensated HF with moderate‐to‐severe left ventricular dysfunction. This recommendation is particularly relevant for patients with a recent onset of clinical syndrome who are refractory to standard HF treatment.^3^, ^4^ In contrast to the widespread opinion that EMB is a hazardous procedure, a number of studies have shown that EMB is relatively safe with major complication rates of 1–3%.^5^, ^6^, ^7^, ^8^, ^9^ Yet, a key problem remains the reliable interpretation of LV‐EMB in regard to its therapeutic consequences, the interobserver variability and potential sampling errors.^10^, ^11^, ^12^, ^13^, ^14^, ^15^ The role of EMB in estimating the prognosis of DCM patients is scarcely understood and was not studied in larger sample sets so far.

In DCM, pathological myocardial remodelling is associated with worse outcome of affected patients. Myocardial fibrosis and extracellular matrix expansion occur during remodelling and are direct consequences of neurohumoral pathway activation, cardiomyocyte apoptosis, and active cardiomyocyte‐fibroblast signalling.^16^, ^17^, ^18^, ^19^ On a molecular level, novel biomarkers aim to assess fibrotic remodelling^20^ and imaging tools, such as cardiac magnetic resonance imaging (MRI), are capable of detecting scars as well as larger fibrotic formations using gadolinium contrast agent or T1 mapping.^21^, ^22^ Although there are considerable advances in these non‐invasive diagnostic tools, the direct assessment of the underlying pathological substrate can identify the individual causes (e.g. viral replication) and may allow prediction of near or long‐term cardiac events.

The aim of the current study was to assess the capability of EMB in estimating the prognosis of DCM patients. To do so, we used digital histopathology and automated image processing in a large‐scale, longitudinal cohort of DCM patients.

## Methods

### Patient enrolment, follow‐up, and endpoint definition

The study was conducted in accordance with the Declaration of Helsinki. The ethics committee of the University Hospital Heidelberg approved the study and all patients had given informed written consent at the time of biopsy taking. Altogether, data were collected from 524 consecutive patients who presented to a tertiary university centre (Heidelberg, Germany) with clinically suspected non‐ischaemic DCM for invasive diagnostic work‐up over a period of 10 years. Patients with hypertensive or congenital heart diseases, as well as those with significant coronary artery disease or primary valvular diseases were not included in this cohort. Patients with suspected acute myocarditis, sarcoidosis, or storage disease and those with LV‐EMB with insufficient quality were also excluded. Samples with insufficient quality for analysis were defined as those with tissue dimensions <2 mm^2^, inadequate fixation, missing Masson's Trichrome (Tri) or Acid Fuchsin Orange (AFOG) staining, or staining artefacts that impaired automated quantification of collagen volume fraction (CVF).

Relevant clinical and imaging data, including gender, body mass index (BMI), New York Heart Association (NYHA) functional class, cardiovascular risk factors, medications, blood test results, 6‐min walk test, spiroergometry, electrocardiography, echocardiography, cardiac MRI, and cardiac device therapy, were collected from in‐house records and patient registries. Clinical data at the time of biopsy were obtained retrospectively, as all biopsies had already been performed at the time of study cohort formation. Data sources included the HeLuMa database of the Heart Failure Registry Heidelberg‐Ludwigshafen‐Mannheim and the clinical data storage system IS‐H. Two endpoints were defined: (i) composite endpoint of HF‐related death, SCD, aborted SCD (appropriate implantable cardioverter‐defibrillator [ICD] shock, reported sustained ventricular tachycardia, or cardiopulmonary resuscitation), or cardiac transplantation, and (ii) all‐cause mortality. Patient events were identified through longitudinal follow‐up using the aforementioned databases, or telephone contact. The composite endpoint was reached at the occurrence of the first event.

### Histopathological preparation and collagen volume fraction quantification

**Figure 1:**
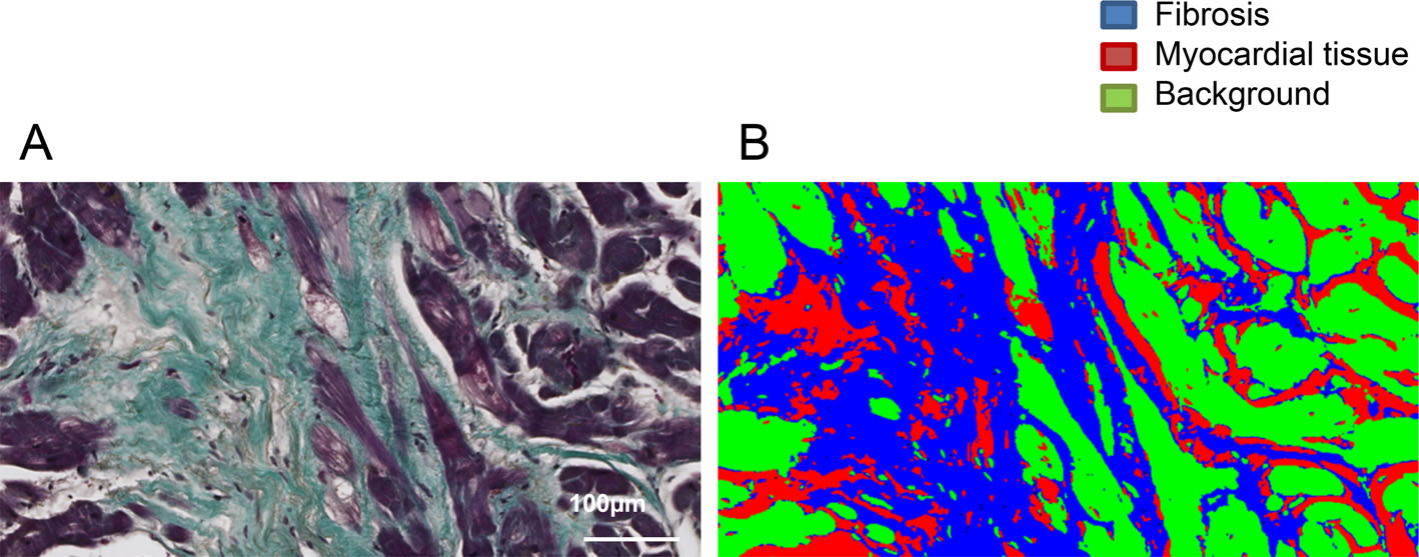
Quantification of collagen volume fraction using automatic image processing algorithms: histological sections are stained using Trichrome or Acid Fuchsin Orange (AFOG) staining methods (*A*). Pixel wise classification of the whole slide is done using a linear Bayesian classifier in RGB colour space for best contrasting (*B*). Collagen volume fraction was calculated as blue (fibrosis) area relative to total tissue area (red + blue), excluding background (green).

Endomyocardial biopsies were taken around the apical to free‐wall of the left ventricle according to a standard operating procedure. Samples were rinsed in NaCl and fixed immediately in 10% neutral buffered formalin, embedded in paraffin, and stained using Tri or AFOG staining methods. Histological sections were automatically imaged in a 20× magnification (resolution: 0.46 μm/pixel) using the Hamamatsu NanoZoomer 2.0‐HT Scan System (Hamamatsu Photonics, Hamamatsu, Japan). For the scanning of the glass slides, the slide scanner automatically detects the region of interest that contains the tissue and also determines automatically a valid focal plane for scanning. The image processing algorithms have been developed using VisiomorphDP version 4.5.1.324 (Visiopharm, Hoersholm, Denmark). Image processing was performed in three distinguished steps: (i) automatic region of interest detection using thresholding methods in the RGB colour space.^23^ The results of this step are the tissue regions separated from the background for further analysis. (ii) Pixelwise classification of the whole slide by using a linear Bayesian classifier in RGB colour space. The classifier was trained by manually marking the different tissue types, the normal cardiomyocytes and the CVF, as well as the background areas within the region of interest.^24^ After training, the classifier was able to separate the processed area into one of these three subcategories. (iii) As a post‐processing step, inappropriate regions (too small tissue fragments, staining artefacts, dust particles) were removed manually or by using morphological operations like opening and closing.^25^ The total area of all available biopsy samples from each patient was measured to ensure a comprehensive and standardized fibrosis assessment. The image processing steps provided output variables representing the areas of different tissue subcategories, including cardiomyocytes, CVF, and background area (*Figure* *1*). CVF was quantified as the ratio of fibrosis relative to the total tissue area and expressed as a percentage using the following formula:

$$\begin{array}{ll} & \text{Collagen volume fraction}\;\left(\%\right)\\ & \;=\frac{\text{Total fibrosis area}\;\left({\mathrm{mm}}^{2}\right)}{\text{Total muscle area}\;\left({\mathrm{mm}}^{2}\right)+\text{Total fibrosis area}\;\left({\mathrm{mm}}^{2}\right)} \end{array}$$

By incorporating all available biopsy samples per patient, this approach minimized sampling bias and ensured a robust evaluation of myocardial fibrosis. The quality of the algorithm to estimate the extent of CVF was controlled by two pathologists blinded to patients' identity or condition.

### Cardiac magnetic resonance imaging as a comparative imaging technique in fibrosis assessment

To further evaluate their clinical phenotype, 274 patients of the cohort (52.3%) underwent cardiac MRI examinations (1.5 T, 32‐channel RF coil, Philips Achieva) within 6 months after their first visit at the centre. Standard examination protocols were performed in all cases as previously described.^26^ Twelve short‐axis slices and 2‐, 3‐ and 4‐ chamber long‐axis projections of the left ventricle were obtained. Post‐acquisition image analysis was performed using cvi42 (Circle Cardiovascular Imaging, Calgary, Canada). The short‐axis sequences of the left ventricle were segmented manually for endo‐ and epicardial borders at end‐diastole and end‐systole phases. The atrioventricular plane defined the basal boundary of the left ventricle and the papillary muscles were excluded in all analyses. Myocardial function, volume and mass were assessed using standard protocols. Gadobutrol (Gadovist, Bayer HealthCare Pharmaceuticals) or Gd‐DTPA (Magnograph®) through intravenous administration was used for late gadolinium enhancement (LGE) imaging purposes in 124 patients. Spectral presaturation with inversion recovery (SPIR) short‐axis sequences were obtained for all patients. Two independent cardiologists, who were blinded to all clinical data and outcomes, performed the assessment of the presence and location of LGE. LGE mass in grams was calculated using the estimated area of LGE in the short axis using the five standard deviation protocols.^27^ Mid‐wall LGE was defined as a visible enhancement in the intramural or subepicardial layers in two phase‐encoding directions and two orthogonal views.^28^

### Statistical analysis

Clinical and imaging characteristics as well as associations between left ventricular CVF (LV‐CVF) (%) and known predictors of HF outcome (age, BMI, left ventricular ejection fraction [LVEF], NT‐proBNP, dyspnoea, renal function, and diabetes) were analysed using standard descriptive statistics. The potential influence of these predictors on HF associated events was then assessed in simple (single‐predictor) Cox regression models and in models additionally adjusted for LV‐CVF. A multivariable, mutually adjusted model was fitted with all predictors that were associated with the outcome at the 10% significance level, and backwards elimination was performed until all remaining variables in the model were associated with the outcome at the 5% level. Possible non‐linearity of effects was evaluated by restricted cubic regression splines in univariable models. If non‐linear effects statistically significantly improved the model fit, they were retained. Multiple imputation using bootstrap and predictive mean matching was applied to account for missing values. Fifty datasets were generated for each regression model and eight variables were used for imputation (LV‐CVF, age, BMI, LVEF, log[NT‐proBNP], dyspnoea, renal function, and diabetes). For comparison of non‐nested models, the Akaike information criterion (AIC) was applied. For the AIC, lower values indicate better model fits under consideration of model complexity (number of included variables). The regression analyses were repeated in an analogous manner for all‐cause mortality as the other endpoint.

An optimal cutpoint for discrimination between patients with a lower and higher risk of HF‐associated events was determined. For this purpose, 91 dichotomous grouping variables based on LV‐CVF were generated (<5% vs. ≥5%, <6% vs. ≥6%, <7% vs. ≥7%, …, <93% vs. ≥93%, <94% vs. ≥94, <95% vs. ≥95%). With each of these a log‐rank test was computed and the distribution of *p*‐values over the range of cutpoints was explored. The lowest *p*‐value indicated an optimal cutpoint. Clinical and imaging characteristics were then compared across the two groups formed by this cutpoint.

All statistical tests were two‐sided and *p*‐values <0.05 were considered statistically significant. Since multiple tests were performed and no adjustment for multiplicity was applied, *p*‐values can be interpreted only in a descriptive manner. The statistical analyses were performed in R, using the packages ‘rms’, ‘Hmisc’ and ‘ggplot2’.^29^, ^30^, ^31^

## Results

### Patient demographics and clinical profiles

In total, 524 consecutive DCM patients (mean age 54 ± 13.4 years; 67.4% male) were included in this study. Patients' age, BMI, LVEF, and log(NT‐proBNP) were distributed as shown in online supplementary *Figure* *S1*. Seventy‐three (13.9%) patients had a definitive positive family history for DCM, while 86.1% were classified as sporadic DCM cases without additional affected family members. High‐sensitivity cardiac troponin T (hsTnT) was increased (median 16.0 [7.0–35.0] pg/ml), and NT‐proBNP was strongly elevated (median 655.0 [218.0–2253.0] ng/L) in the cohort. The mean LVEF measured by transthoracic echocardiography was 31.3 ± 12.5%. About 70% of patients were compensated at the time of presentation with a NYHA functional class I or II and could walk 499 ± 127 m in the 6 min‐walk test. The detailed baseline characteristics of the study cohort are listed in *Table* *1*.

**Table 1:** Clinical and imaging characteristics at the time of left ventricular endomyocardial biopsy

Characteristics	All patients (*n* = 524)	LV‐CVF ≤32% (*n* = 260)	LV‐CVF >32% (*n* = 264)	*p*‐value*
Age, years, mean (SD)	54.0 (13.4)	53.2 (14.2)	54.7 (12.6)	0.23
Male sex, *n* (%)	353 (67.4)	180 (69.2)	173 (65.5)	0.37
BMI, kg/m^2^, mean (SD)	27.1 (5.7)	27.7 (5.4)	26.5 (5.8)	0.02
Arterial hypertension, *n* (%)	351 (80.0)	183 (82.1)	168 (77.8)	0.26
Diabetes, *n* (%)	72 (17.6)	35 (16.8)	37 (18.5)	0.64
Tobacco smoking, *n* (%)				
Current smoker	95 (28.7)	50 (29.1)	45 (28.3)	
Never smoker	153 (46.2)	77 (44.8)	76 (47.8)	0.84
Quit smoking	83 (25.1)	45 (26.2)	38 (23.9)	
Alcohol excess^a^, *n* (%)	9 (6.2)	3 (3.9)	6 (8.8)	0.21
Family history of DCM, *n* (%)	73 (13.9)	36 (18.6)	37 (21.4)	0.50
Lipid profile				
Total cholesterol, mg/dl, mean (SD)	187.6 (47.3)	190.2 (43.6)	185.3 (50.5)	0.46
High‐density lipoprotein, mg/dl, mean (SD)	48.4 (16.1)	47.2 (15.1)	49.4 (16.9)	0.33
Low‐density lipoprotein, mg/dl, mean (SD)	109.7 (33.6)	113.5 (31.0)	106.6 (35.4)	0.16
Triglyceride, mg/dl, mean (SD)	140.7 (85.1)	146.3 (78.8)	135.0 (91.9)	0.55
White blood cell count, /nl, mean (SD)	8.3 (3.0)	8.4 (3.1)	8.2 (2.9)	0.53
Haemoglobin, g/dl, mean (SD)	13.9 (1.7)	14.0 (1.8)	13.7 (1.6)	0.20
Serum creatinine, mg/dl, mean (SD)	1.0 (0.6)	1.0 (0.6)	1.1 (0.6)	0.33
NT‐proBNP, ng/L, median (IQR)	655.0 [218.0; 2253.0]	535.0 [168.0; 1805.0]	860.8 [249.5; 3239.0]	0.02
hs‐TnT, pg/ml, median (IQR)	16.0 [7.0; 35.0]	14.0 [5.0; 32.5]	18.0 [10.0; 38.0]	0.09
Heart rate, bpm, mean (SD)	81.7 (21.2)	80.4 (22.0)	83.1 (20.5)	0.53
Left bundle branch block, *n* (%)	19 (18.6)	6 (11.1)	13 (27.1)	0.04
Blood pressure, mmHg, mean (SD)				
Systolic	127.6 (19.8)	129.2 (18.8)	125.8 (20.9)	0.44
Diastolic	76.9 (13.0)	78.3 (12.3)	75.3 (13.9)	0.31
Dyspnoea, *n* (%)				
NYHA class				
I	85 (40.3)	45 (42.1)	40 (38.5)	
II	54 (25.6)	31 (29.0)	23 (22.1)	0.14
III	36 (17.1)	19 (17.8)	17 (16.4)	
IV	36 (17.1)	12 (11.2)	24 (23.1)	
6MWT, m, mean (SD)	498.9 (127.1)	492.4 (102.6)	504.1 (144.8)	0.67
VO_2_ max, ml/kg/min, mean (SD)	17.7 (5.7)	17.4 (5.4)	17.8 (6.0)	0.79
Transthoracic echocardiography				
LV ejection fraction, %, mean (SD)	31.2 (17.5)	33.6 (18.5)	28.8 (16.1)	<0.001
LV diastolic dysfunction, *n* (%)	34 (40.5)	20 (40.8)	14 (40)	0.94
GLS, −%	12.7 ± 4.3	13.5 ± 4.3	11.7 ± 4.2	0.001
Cardiac MRI data, mean (SD)				
LV ejection fraction, %	31.3 (12.5)	33.7 (13.2)	28.7 (11.3)	<0.001
LV stroke volume, ml	88.0 (31.1)	90.7 (25.9)	86.2 (34.3)	0.49
LVESV index, ml/m^2^	80.3 (46.1)	76.1 (41.4)	84.3 (50.1)	0.21
LVEDV index, ml/m^2^	123.3 (44.8)	119.2 (40.5)	127.2 (48.3)	0.21
LVESD index, mm/m^2^	24.7 (6.4)	23.9 (5.9)	25.5 (6.7)	0.08
LVEDD index, mm/m^2^	31.0 (5.2)	30.3 (4.8)	31.6 (5.5)	0.07
LV mass index, g/m^2^	62.4 (21.2)	60.5 (19.6)	64.7 (23.0)	0.27
Septum wall thickness, mm	9.7 (2.4)	9.7 (2.2)	9.6 (2.7)	0.74
RVEDD index, mm/m^2^	23.3 (4.3)	23.2 (4.3)	23.4 (4.4)	0.78
LA diameter, mm	39.0 (8.3)	38.1 (7.1)	40.3 (9.7)	0.14
MAPSE, mm	8.6 (3.4)	9.0 (3.2)	8.2 (3.6)	0.22
TAPSE, mm	18.2 (4.8)	18.2 (4.5)	18.2 (5.2)	0.99

6MWT, 6‐min walk test; BMI, body mass index; DCM, dilated cardiomyopathy; GLS, global longitudinal strain; hs‐TnT, high‐sensitivity troponin T; IQR, interquartile range; LA, left atrial; LV, left ventricular; LF‐CVF, left ventricular collagen volume fraction; LVEDD, left ventricular end‐diastolic diameter; LVEDV, left ventricular end‐diastolic volume; LVESD, left ventricular end‐systolic diameter; LVESV, left ventricular end‐systolic volume; MAPSE, mitral annular plane systolic excursion; MRI, magnetic resonance imaging; NYHA, New York Heart Association; NT‐proBNP, N‐terminal pro‐B‐type natriuretic peptide; SD, standard deviation; RVEDD, right ventricular end‐diastolic diameter; TAPSE, tricuspid annular plane systolic excursion; VO_2_, oxygen uptake.

* Calculated using either the *t*‐test or Mann–Whitney U test for comparison of means and medians, respectively, or chi‐squared test for comparisons of categorical variables.

^a^ Defined as consistent intake of 4 or more units/day for men and 3 or more units/day for women.

### Procedure safety: low complication rates in endomyocardial biopsy

For each patient, 3–5 LV‐EMB were analysed for histopathological studies with each sample having an area of 1–2 mm^2^. Left heart catheterization including LV‐EMB was associated with 0.76% major complications and 2.1% minor complications as classified by Chimenti *et al*.^8^ (*Table* *2*). There was no case of death associated with the procedure or any patient suffering from long‐term complications.

**Table 2:** Complications of left ventricular endomyocardial biopsy in the study cohort

Complications	
Major complications	
Perforation with cardiac tamponade	1 (0.19%)
Pericardial effusion without pericardiocentesis	3 (0.57%)
Brain embolization with transient cerebral ischaemia	0
Pulmonary embolization	0
Permanent atrioventricular block	0
Death	0
Overall	4 (0.76%)
Minor complications	
Ventricular or supraventricular transient arrhythmias	7 (1.33%)
Transient heart block	2 (0.38%)
Intramyocardial haematoma	0
Local haematoma	0
Femoral arterial venous fistula	2 (0.38%)
Overall	11 (2.10%)

### Estimating patients' fibrosis burden

Using a series of log‐rank tests, an LV‐CVF extent of 32% was determined as the optimal cutpoint discriminator (online supplementary *Figure* *S2*). This cut‐off closely aligns with the median LV‐CVF in our cohort (32.2%), further supporting its clinical relevance. Patients' characteristics were then compared between the two subgroups with LV‐CVF ≤32% and LV‐CVF >32%. The median of NT‐proBNP was significantly higher in the subgroup with LV‐CVF >32% (*p* = 0.02) and the LVEF was lower (*p* < 0.001). In concordance with their NT‐proBNP levels, patients from the subgroup with LV‐CVF >32% were more frequently discharged with diuretics (*p* = 0.02). A total of 27% of patients with LV‐CVF >32% presented left bundle branch block, whereas this could be observed in only about 11% of those with LV‐CVF ≤32% (*p* = 0.04). Further known prognostic factors of HF such as right ventricular end‐diastolic diameter index (*p* = 0.78), left atrial diameter (*p* = 0.14), and peak oxygen uptake (*p* = 0.79) did not differ significantly between the two subgroups (*Table* *1*).

### Collagen volume fraction as a predictor of heart failure and mortality

Patients were followed up for a median duration of 3.6 years (max 8 years). The study analysed altogether 2084 patient‐years. A total of 62 patients reached the composite endpoint of HF‐associated events with 35 cardiovascular deaths and 55 patients reached the endpoint all‐cause mortality (online supplementary *Figure* *S3*).

**Figure 2:**
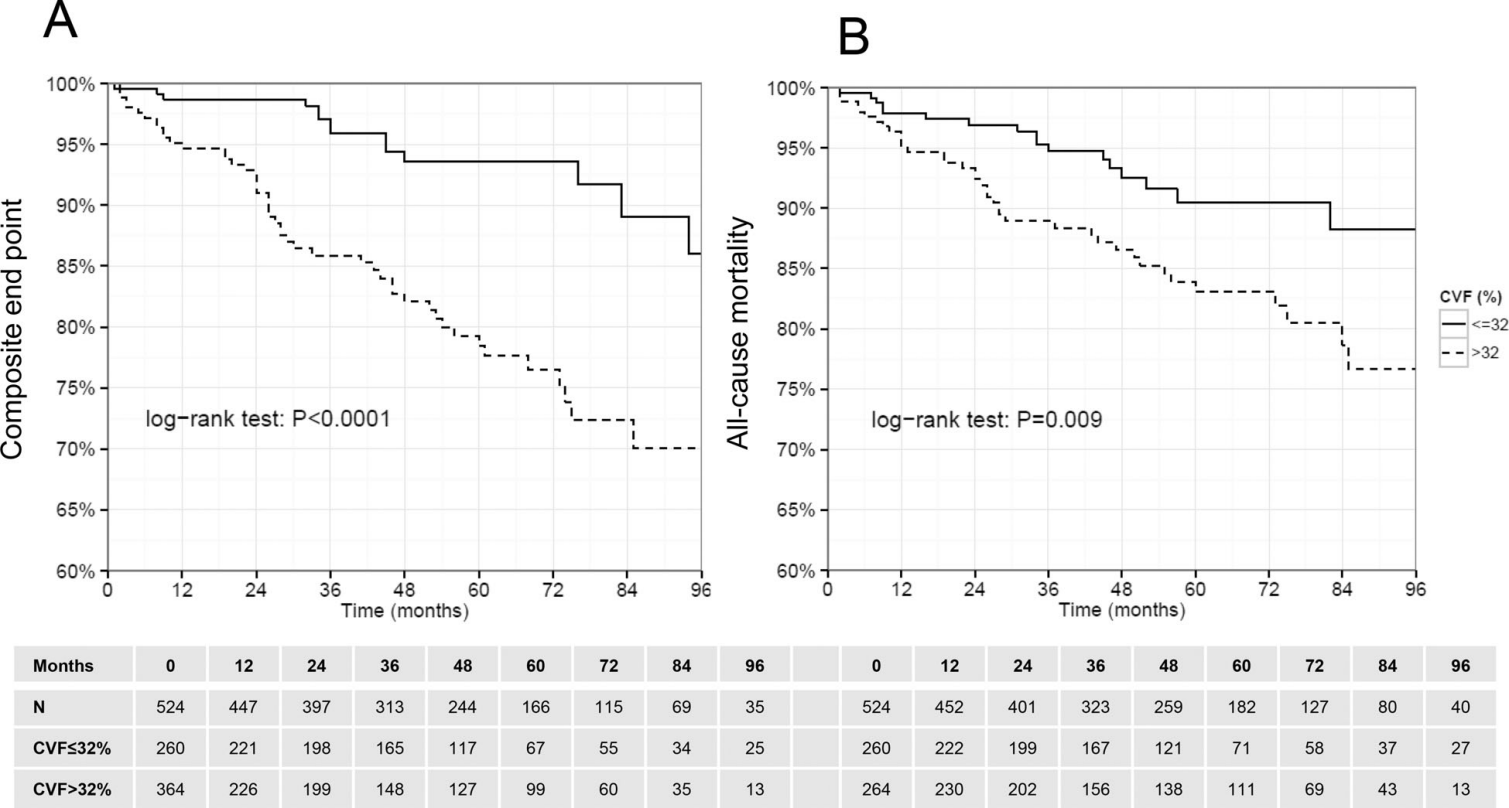
Kaplan–Meier survival estimates of the time to events depending on the extent of left ventricular collagen volume fraction: patients with higher extent of LV‐CVF (>32%) demonstrate a significantly poorer survival rate in composite endpoint (*A*) and all‐cause mortality (*B*).

The extent of LV‐CVF was associated with a significantly higher risk for HF‐associated events (18.2% vs. 5.4%; *p* < 0.0001; *Figure* *2A*) as well as for all‐cause mortality (14.4% vs. 6.5%; *p* = 0.009; *Figure* *2B*). There were only modest associations between the extent of LV‐CVF and other predictors of HF outcome as shown in online supplementary *Table* *S1*.

In univariable Cox regression analysis, LVEF (hazard ratio [HR] 0.87, 95% confidence interval [CI] 0.77–0.99, per 5 percentage points, *p* = 0.03) and log(NT‐proBNP) (HR 1.38, 95% CI 1.14–1.66, *p* < 0.001) were predictors of the endpoint HF‐associated events. Multivariable analysis showed that two‐predictor models that include LV‐CVF outperformed the single‐predictor models, as demonstrated by the lower AIC, indicating that the extent of LV‐CVF is an independent predictor of HF‐associated events and all‐cause mortality (*Table* *3*).

**Table 3:** Predictors of heart failure‐associated events (Cox proportional hazards models)

Variable	Single‐predictor models			Two‐predictor models adjusted for LV‐CVF		
	HR (95% CI)	*p*‐value	AIC	HR (95% CI)	*p*‐value	AIC
Age, years/10	1.19 (0.97–1.45)	0.08	701.4	1.19 (0.98–1.46)	0.08	689.6
BMI	0.94 (0.90–1.00)	0.06	700.9	0.96 (0.91–1.01)	0.12	690.1
LV ejection fraction, %/5	0.87 (0.77–0.99)	0.03	700.2	0.89 (0.78–1.01)	0.09	690.4
Log (NT‐proBNP)	1.38 (1.14–1.66)	<0.001	684.9	2.03 (1.32–3.11)	0.001	675.5
Dyspnoea						
NYHA class						
I	1 (ref.)	0.23	704.5	1 (ref.)	0.42	694.5
II	0.85 (0.37–1.97)			0.88 (0.38–2.02)		
III	1.18 (0.48–2.86)			1.18 (0.48–2.85)		
IV	1.93 (0.90–4.13)			1.72 (0.80–3.72)		
Atrial fibrillation						
No	1 (ref.)	0.58	704.0	1 (ref.)	0.36	691.5
Yes	1.19 (0.65–2.20)			1.33 (0.72–2.47)		
Diabetes						
No	1 (ref.)	0.10	701.5	1 (ref.)	0.11	689.9
Yes	1.70 (0.90–3.20)			1.69 (0.89–3.21)		
Log (creatinine)	1.21 (0.95–1.54)	0.12	529.3	1.23 (0.97–1.56)	0.09	521.8

Multiple imputation was used to account for missing data.

AIC, Akaike information criterion; BMI, body mass index; CI, confidence interval; HR, hazard ratio; LV, left ventricular; LF‐CVF, left ventricular collagen volume fraction; NT‐proBNP, N‐terminal pro‐B‐type natriuretic peptide.

### Ventricular arrhythmias and implantable cardioverter‐defibrillator therapy in relation to fibrosis burden

During follow‐up, sustained ventricular tachycardia/fibrillation occurred in 28 patients, with a higher proportion in those with LV‐CVF >32% (*n* = 20) compared to ≤32% (*n* = 8). Among patients who received an ICD (*n* = 114), appropriate ICD shocks were documented in 11 patients with LV‐CVF >32% and 5 patients with LV‐CVF ≤32%, suggesting a potential link between increased fibrosis burden and arrhythmic risk.

### Associations between cardiac magnetic resonance imaging findings and collagen volume fraction

**Figure 3:**
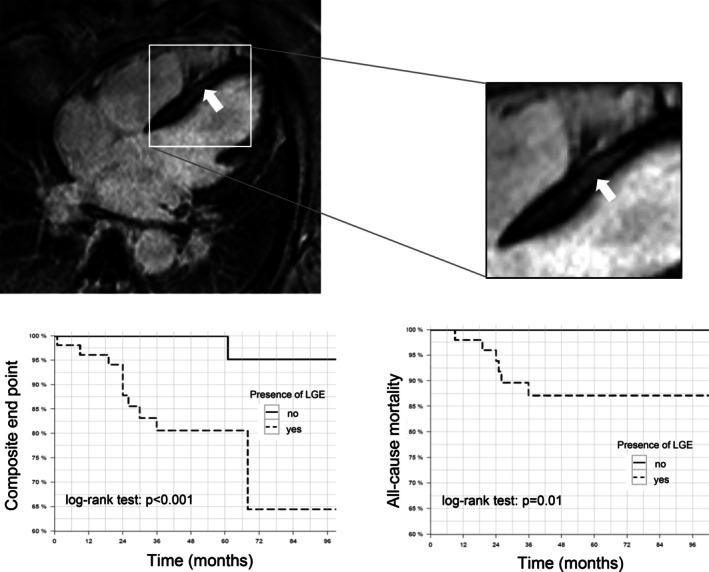
Late gadolinium enhancement on cardiac magnetic resonance imaging (LGE) was quantified using cvi42 software. Presence of LGE correlates with the two unfavorable outcome measures of composite endpoint and all‐cause mortality.

Since fibrosis could also be assessed non‐invasively, we analysed all patients who received intravenous gadolinium during cardiac MRI at the centre within 6 months after myocardial biopsy taking (*n* = 124). Fifty‐five (44.3%) were positive for LGE. The absence or presence of LGE correlated significantly with the defined endpoints (*p* < 0.001 for the composite endpoint and *p* = 0.01 for the endpoint all‐cause mortality) (*Figure* *3*). The extent of LGE did not significantly correlate with the extent of diffuse interstitial LV‐CVF measured by LV‐EMB (*R* = 0.04, 95% CI –0.14 to 0.21, *p* = 0.7 for LGE%[logit]) (online supplementary *Figure* *S4*), suggesting that LGE represents larger, often intramyocardial scar formation.

## Discussion

Despite the therapeutic consequences gained from LV‐EMB in selected cases of viral or inflammatory DCM, its value in clinically stable DCM patients is discussed controversially.^32^ In the era of integrated phenotypic approach for an aetiologically‐guided management as recommended by the latest European Society of Cardiology guidelines,^4^ EMB is, however, an aspiring, complication‐poor diagnostic interventional procedure. Clinically relevant complications of left or right ventricular EMB can be divided into major and minor ones and are estimated at 1–3%, varying with operators' experience, clinical state of patients and aetiology of the underlying cardiac disease.^5^, ^6^, ^7^, ^8^, ^9^ Deaths, mostly as a result of perforation with pericardial tamponade or air embolism, have been reported to be rare events in association with LV‐EMB, showing an incidence of <0.2%.^33^, ^34^ Whereas in other studies only a few specially trained interventionalists performed the biopsies—an approach considered unrealistic in real‐life practice^8^—in the present study, 24 cardiologists performed the EMBs with a low complication rate of 0.76% major events and no deaths or air embolisms. Only one case required pericardiocentesis. These data underline the safety in daily medical practice. All EMB in this study were obtained from the left ventricle. Although right ventricular biopsies are more common in clinical practice, we favour the left ventricular approach, as it may reduce the risk of complications such as tricuspid valve injury, cardiac perforation, and tamponade.^8^, ^35^ However, recent data have demonstrated a positive correlation between fibrosis detected in right ventricular biopsies and left ventricular fibrosis in explanted hearts, suggesting that from the perspective of fibrotic burden assessment, both biopsy sites may provide comparable information.^36^

Myocardial fibrosis has already been suggested to be an important predictor of adverse outcome and possible target for future interventional trials.^20^, ^37^ Reactive or reparative myocardial fibrosis can occur in different patterns even in those DCM patients with normal or only moderately reduced systolic function (left ventricular non‐DCM), associated with an increased risk for ventricular arrhythmias and SCD.^38^, ^39^, ^40^ It is characterized by accumulation of collagens and other components of the extracellular matrix such as elastic fibers, glycoproteins, proteoglycans, growth factors, cytokines, and proteases and can be estimated by CVF.^41^, ^42^ Thus, increased CVF through increased production or decreased degradation of collagen is, regardless of the aetiology, associated with increased mechanical stiffness and deterioration of diastolic and ultimately systolic function. Furthermore, it may disrupt the electrical coupling between layers of cardiomyocytes, building a substrate for and predisposing to arrhythmogenicity and SCD.^19^, ^43^ It seems thus appropriate to stratify patients according to their fibrotic burden.

As standard diagnostic tool, echocardiography can be used to robustly assess left ventricular diastolic function. However, this does not necessarily reflect cardiac fibrosis, as diastolic function also depends on filling pressures and active myocyte relaxation.^44^ Other non‐invasive diagnostic tools such as cardiac MRI might be capable of estimating cardiac fibrosis and be preferred by many cardiologists. LGE on cardiac MRI is associated with a ~2.5‐fold higher 5‐year mortality and with higher risk of SCD, worsening HF, and need for cardiac transplantation.^45^ However, cohort studies of patients with cardiomyopathies reveal that the agreement between cardiac MRI and EMB findings is only partial, highlighting the complementary nature of these procedures in diagnostic assessment.^46^, ^47^ While cardiac MRI provides detailed imaging and functional information, EMB offers histopathological insights that are crucial for definitive diagnosis in certain conditions.^48^, ^49^ By integrating the strengths of both cardiac MRI and EMB, clinicians can achieve a more comprehensive diagnostic evaluation, ultimately improving patient outcomes.

Currently, treatment of DCM including ICD implantation is predominantly based on functional variables such as LVEF. Reduced LVEF (≤35%), NYHA functional class, as well as its worsening have been ascribed prognostic importance in DCM patients.^50^, ^51^ However, these parameters are not impeccable in non‐ischaemic DCM, which can be in part compensated by adding information of molecular biomarkers such as B‐type natriuretic peptide or troponins.^52^, ^53^ In this study, we could corroborate the hypothesis that the extent of CVF in LV‐EMB is an independent predictor of outcome in patients with DCM. Saito *et al*.^13^ previously delineated a prognostic role for ultrastructural endomyocardial changes in DCM patients, showing that both focal derangement and diffuse myofilament lysis in electron microscopy were independent predictors for a composite HF endpoint (readmission due to HF and HF death). The approach presented in this study is technically straightforward, enables robust quantification of myocardial fibrosis—the gold standard for its assessment^54^—and may therefore be well suited to be integrated into the routine histological work‐up of EMB, whenever taken.

### Study limitations

The here described cohort consisted of DCM patients who were referred to our centre and can thus be subject to referral bias. Some of the potential limitations of histopathological evaluations of EMB have already been mentioned above. Moreover, depending on the site of biopsy the extent of CVF can vary.^55^ For instance, fibrotic tissue can be overestimated if the biopsy originates from the site of previous sample taking. This could though be ruled out in our patients as all underwent LV‐EMB for the first time. An in‐house standard for the interventional procedure and the usage of the same bioptome model and a femoral access site over the whole study period should eliminate some sources of bias as well. The limited availability of T1 mapping‐based cardiac MRI examinations in our cohort prevented their inclusion in the analysis. This is primarily due to the early recruitment period of the cohort, which enabled long‐term follow‐up but preceded the routine clinical implementation of T1 mapping. Nevertheless, T1 mapping and extracellular volume quantification represent promising non‐invasive approaches for detecting diffuse myocardial fibrosis and are being extensively investigated as surrogates of cardiac CVF.^56^ This was a single‐centre study and the findings need to be validated in a multicentre setting.

## Conclusions

Left ventricular EMB is a safe diagnostic tool in suspected DCM. Using automated methods to estimate the extent of CVF as developed here can decrease limitations such as interobserver variability. Our findings demonstrate that the extent of CVF, assessed via LV‐EMB, is an independent prognostic factor in patients with DCM (*Graphical Abstract*). When EMB is performed for diagnostic purposes, histological assessment of myocardial fibrosis should be integrated into risk stratification. Patients with elevated LV‐CVF may benefit from closer clinical monitoring due to a higher risk for arrhythmias and disease progression. In addition, CVF quantification offers a valuable biomarker for future cardiovascular research aimed at evaluating antifibrotic therapies and guiding personalized treatment strategies.

## Supporting information


**Appendix S1.** Supporting Information.
