# Supplementary material for: Safety and prognostic value of left ventricular endomyocardial biopsy in dilated cardiomyopathy
Source: Eur J Heart Fail. 2025 Sep 19;27(12):3029–39. doi: 10.1002/ejhf.70019 (PMC12803607; doi:10.1002/ejhf.70019)
Supplement: Supplementary file 1 — Appendix S1. Supporting Information. [file EJHF-27-3029-s001.zip › ejhf70019-sup-0001-Appendix S1/ejhf70019-sup-0001-Figures.pptx]

## Slide 1
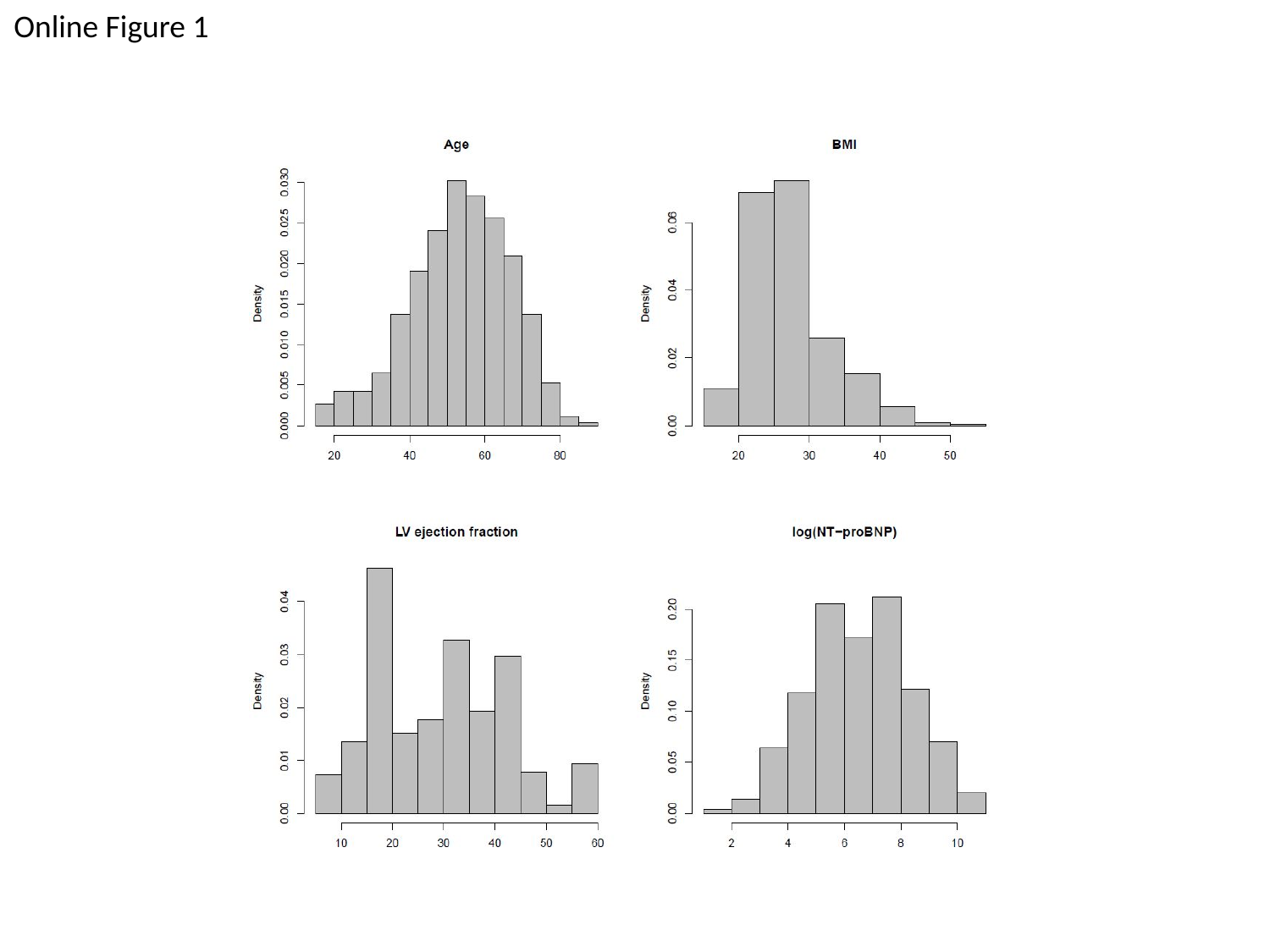

Online Figure 1

## Slide 2
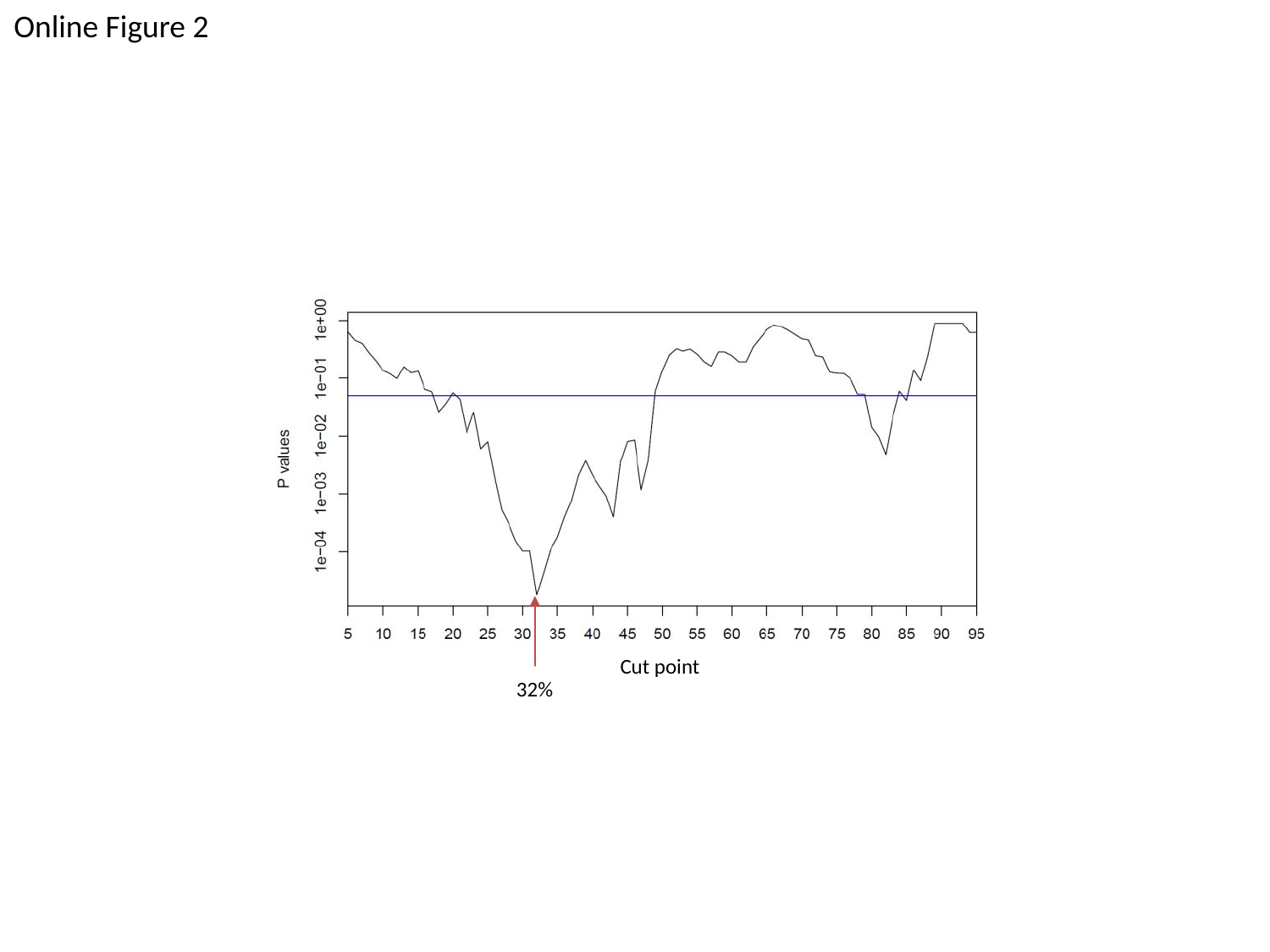

Online Figure 2
Cut point
32%

## Slide 3
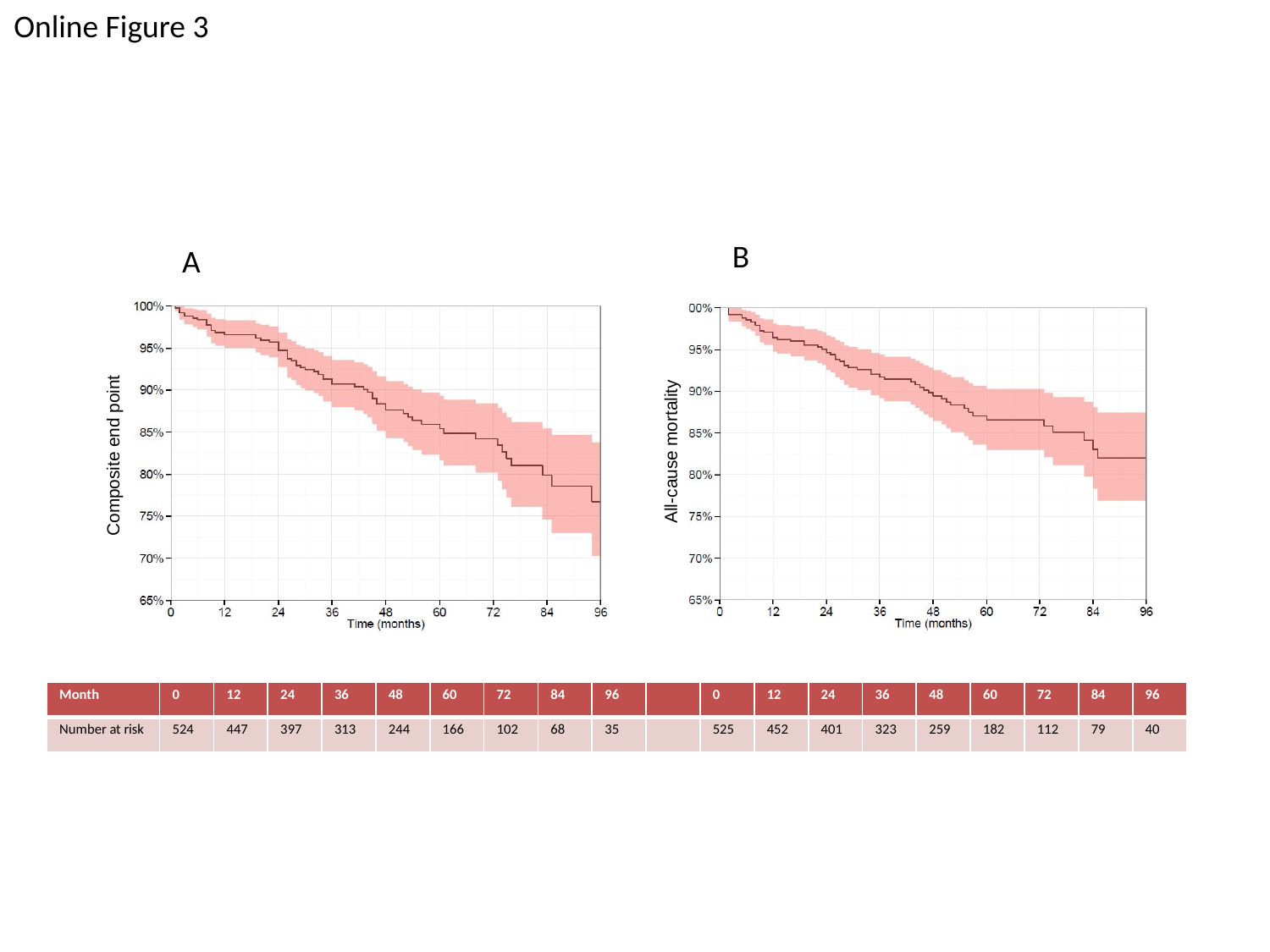

Online Figure 3
B
A
All-cause mortality
Composite end point
| Month | 0 | 12 | 24 | 36 | 48 | 60 | 72 | 84 | 96 | | 0 | 12 | 24 | 36 | 48 | 60 | 72 | 84 | 96 |
| --- | --- | --- | --- | --- | --- | --- | --- | --- | --- | --- | --- | --- | --- | --- | --- | --- | --- | --- | --- |
| Number at risk | 524 | 447 | 397 | 313 | 244 | 166 | 102 | 68 | 35 | | 525 | 452 | 401 | 323 | 259 | 182 | 112 | 79 | 40 |

## Slide 4
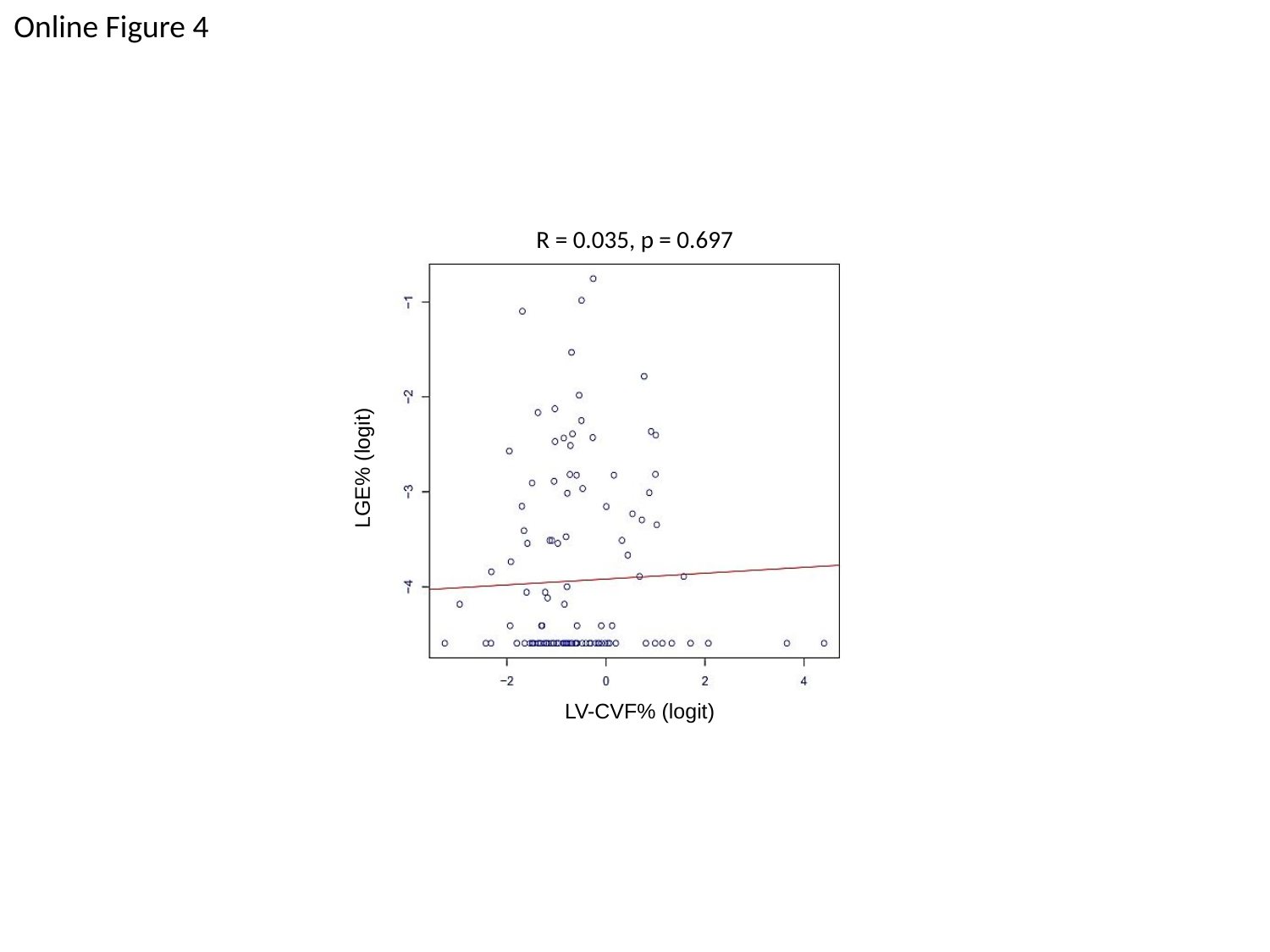

Online Figure 4
R = 0.035, p = 0.697
LGE% (logit)
LV-CVF% (logit)
