# Supplementary material for: Safety and prognostic value of left ventricular endomyocardial biopsy in dilated cardiomyopathy
Source: Eur J Heart Fail. 2025 Sep 19;27(12):3029–39. doi: 10.1002/ejhf.70019 (PMC12803607; doi:10.1002/ejhf.70019)
Supplement: Supplementary file 1 — Appendix S1. Supporting Information. [file EJHF-27-3029-s001.zip › ejhf70019-sup-0001-Appendix S1/ejhf70019-sup-0002-Tables.docx]

**Online Table 1: Associations between LV-CVF (%) and other predictors of HF outcome**

| **Variable** | **N** | **Pearson correlation coefficient (95%-CI)** | | ***p* value** |
| --- | --- | --- | --- | --- |
| Age, years | 524 | -0.03 (-0.11, 0.06) | | 0.54 |
| BMI | 471 | -0.08 (-0.17, 0.01) | | 0.07 |
| LV ejection fraction, % | 385 | -0.11 (0.21, -0.01) | | 0.03 |
| log(NT-proBNP) | 524 | 0.05 (-0.07, 0.16) | | 0.40 |
| Log(Creatinine) | 524 | 0.01 (-0.09,0.11) | | 0.90 |
| **Variable** | **N** | **Mean (95%-CI)** | **Difference (95%-CI)** | ***p* value^a^** |
| Dyspnoea |  |  |  |  |
| NYHA I-II | 139 | 34.8 (31.4, 38.2) | -4.8 (-10.7, 1.1) | 0.11 |
| NYHA III-IV | 72 | 39.6 (34.8, 44.5) |  |  |
| Atrial fibrillation |  |  |  |  |
| No | 362 | 35.0 (30.5, 39.6) | -3.0 (-8.0, 2.0) | 0.23 |
| Yes | 96 | 38.1 (35.9, 40.2) |  |  |
| ^a^ Based on Welch two-sample t test. Abbreviations: BMI, body mass index; NYHA, New York Heart Association. | | | | |
